# Mechanistic Aspects of the Electrochemical Oxidation of Aliphatic Amines and Aniline Derivatives

**DOI:** 10.3390/molecules28020471

**Published:** 2023-01-04

**Authors:** Ashwin K. V. Mruthunjaya, Angel A. J. Torriero

**Affiliations:** School of Life and Environmental Sciences, Deakin University, Burwood VIC 3125, Australia

**Keywords:** amines, aniline, anodic oxidation, catalysis, mechanism, electrolysis

## Abstract

The electrochemical oxidation of amines is an essential alternative to the conventional chemical transformation that provides critical routes for synthesising and modifying a wide range of chemically useful molecules, including pharmaceuticals and agrochemicals. As a result, the anodic reactivity of these compounds has been extensively researched over the past seven decades. However, the different mechanistic aspects of the electrochemical oxidation of amines have never been discussed from a comprehensive and general point of view. This review examines the oxidation mechanism of aliphatic amines, amides, aniline and aniline derivatives, carbamates, and lactams, either directly oxidised at different electrode surfaces or indirectly oxidised by a reversible redox molecule, in which the reactive form was generated in situ. The mechanisms are compared and simplified to understand all possible pathways for the oxidation of amines using only a few general mechanisms. Examples of the application of these oxidation reactions are also provided.

## 1. Introduction

A normal synthetic reaction implies the attack of a nucleophile on an electrophilic centre, with the reaction between molecules of similar polarity being considered inviable. In this scenario, the inversion of the polarity of one of those molecules is required, which is not an easy task in conventional organic synthesis. However, it is commonly realised in electrochemical organic synthesis, making possible a large variety of reactions [1,2].

Amines are a family of chemical compounds that share as a common feature the presence of at least one nitrogen atom whose hybridisation depends on the structure of the molecule. For example, aliphatic amines contain sp^3^-hybridised nitrogen atoms. This hybridisation lies between sp^3^ and sp^2^ when the amine is part of a resonance structure. Alternatively, it shows sp^2^-hybridisation when forming part of a heterocycle. In all cases, the amines contain a lone pair of electrons in the unbounded orbital, which is responsible for their relatively easy electrochemical oxidation.

Due to the large variety of amine-containing molecules available and extensive research on them over the past seven decades, it is impossible to discuss and cite all work performed in this area in a single paper. Nevertheless, several review articles and book chapters have been published summarising the impressive advances in this field over the years [3,4,5,6,7,8]. However, a detailed discussion of the different mechanistic aspects of the electrochemical oxidation of amines is still elusive. Therefore, this manuscript focuses on the comprehensive discussion of the oxidation mechanism of aliphatic amines, amides, aniline and its derivatives, and carbamates and lactams, the respective similarities and differences between their mechanisms, and catalysed electrochemical oxidations. Examples of the application of these oxidation reactions are also provided.

## 2. Aliphatic Amines

The potential at which aliphatic amines can be electrochemically oxidised depends on their structure (Table 1), with secondary or tertiary amines being easier to oxidise than primary amines. Nevertheless, the general mechanism for the electrochemical oxidation of simple aliphatic amines is the same and independent of the number of organic substituents attached to the nitrogen atom [9,10,11,12,13,14,15]. Upon the oxidation of a tertiary amine, the overall reaction provides a secondary amine, an aldehyde, and protons. These protons protonate the starting amine or the secondary one (product from this reaction) to give an electrochemically inactive ammonium ion, and the reaction consumes one electron per starting molecule [10,13,16,17,18]. Similarly, the oxidation of a secondary amine produces a primary ammonium ion as the product, while the oxidation of primary amines forms ammonia [19].

The one-electron oxidation reaction starts with the amine oxidation to the respective radical cation (Equation (1)), which deprotonates to give a radical at the α-carbon connected to the nitrogen atom (Equation (2)).

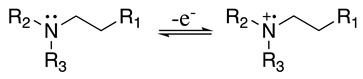
(1)

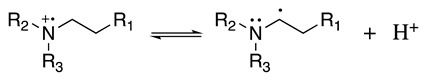
(2)

This radical can be involved in a disproportionation process to yield the starting amine and an enamine (**1**, Equation (3)) or be involved in a second oxidation step to produce an iminium cation (**2**, Equation (4)). Independently of this, it is expected that **1** and **2** be in equilibrium (Equation (5)), as the enamine would be a stronger base than the starting saturated amine [13]. Intermediate **2** is also formed during the oxidation of alicyclic amines, such as piperidine, piperazine, and their derivatives [29].

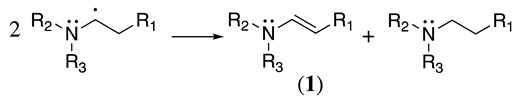
(3)

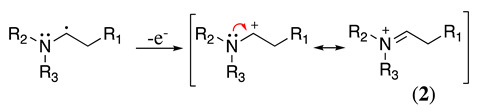
(4)

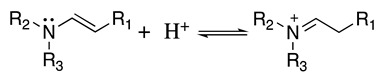
(5)

It is important to highlight that although the mechanism postulates the consumption of two electrons, coulometry generally shows the consumption of one electron per molecule of starting amine because an extra molecule is inactivated by protonation.

Because of its positive charge, **2** is a better electron acceptor than a ketone carbonyl. Thus, any weak or strong nucleophile can react with **2** to form various products. For example, Equations (6) and (7) show the reaction of **2** in the presence of water. It begins with the nucleophilic addition of water to the iminium group (Equation (6)), followed by the transfer of a proton from oxygen to nitrogen to yield the protonated amino alcohol **3** (or carbinolamine), which converts the amine into a better leaving group. Next, the E1-like loss of amine produces a protonated aldehyde (Equation (7)). Finally, the loss of a proton from oxygen gives the final aldehyde and a quaternary ammonium product (Equation (7)).

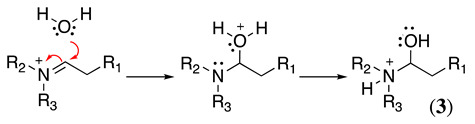
(6)

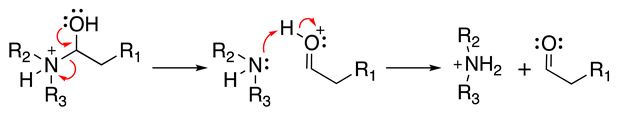
(7)

Ross considered that the hydrolysis reaction follows a concerted or two-step base-catalysed mechanism (Equation (8)) [30], which produces the aldehyde in the enolic form and the amine. Then, keto-enol tautomerisation produces the final aldehyde product. However, this is not ubiquitous, and the following evidence confirmed that Equations (6) and (7) are more appropriate in specific situations [17]:

(1) Equation (8) cannot explain the demethylation of trimethylamine or other methylated amines.

(2) Experimental evidence confirmed that the reaction produces an ammonium ion and not an amine.

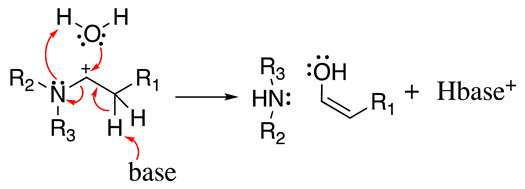
(8)

Alternatively, if the anodic oxidation of amines is performed in the presence of methanol, the methoxylation of the amine-containing molecule is observed. For example, the methoxylation reaction of N,N-dimethylbenzylamine was reported [14,31,32,33]. Based on the previous mechanism, the equivalent molecule of intermediate **2** (Equation (4)) reacts with methanol. However, the molecule offers two possible intermediate **2** (**2a** and **2b**):

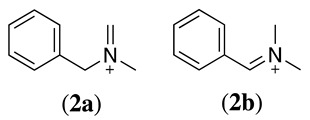

It was observed that **2a** is of greatest preference (methyl attack/benzyl attack = 10) as the methyl hydrogens are more reactive than the methylene hydrogens [31]. Equation (9) shows this reaction and the final product obtained in a larger yield (**4**). The demethylation product N-methylbenzylamine (**5**) and 1-methoxy-N,N-dimethyl-1-phenylmethanamine (**6**) were also obtained as secondary products in the same reaction [31].

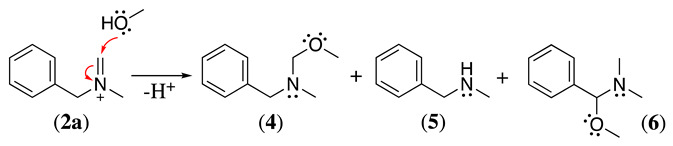
(9)

The electrochemical oxidation of benzylamine in acetonitrile at a stainless-steel mesh anode in the presence of 0.1 M tetrabutylammonium perchlorate as the supporting electrolyte was studied [34]. Due to the absence of methyl groups, the formation of an intermediate equivalent to **2b** occurs, which reacts with starting material (Equation (10)), resulting in the formation of N-benzylidenebenzylamine (**7**) and a small percentage of benzonitrile [34].

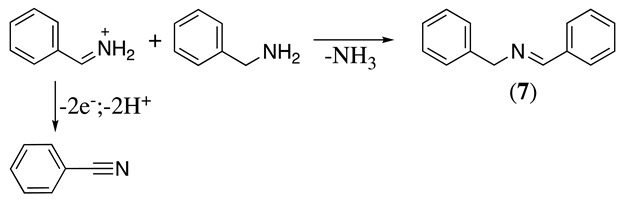
(10)

A similar situation to that described above for N,N-dimethylbenzylamine was observed in our group during the electrochemical oxidation of N,N-dicyclohexylmethylamine, N,N-dimethylcyclohexylamine, and N,N-dicyclohexylamine [17]. Using N,N-dicyclohexylmethylamine as an example, the initial oxidation affords a radical cation (Equation (1)), which can deprotonate following two possible paths to give a radical. One pathway is the formation of cyclohexyl radical **8** (Equation (11)), and the second option is the formation of methylene radical **9** (Equation (12)). From the electrolysis results of N,N-dicyclohexylmethylamine and N,N-dimethylcyclohexylamine, it was possible to observe that the formation of radical **9** is preferential over that of radical **8**.

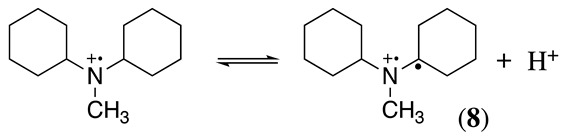
(11)

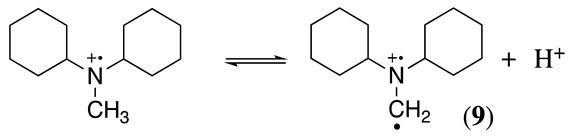
(12)

However, in the absence of methyl groups, the formation of radical **8** occurs, as was observed with N,N-dicyclohexylamine. It seems worthwhile to point out that in the case of radical **9**, an enamine intermediate cannot be formed. Nevertheless, the demethylation process still takes place. Water molecules in the organic solvent reacted with the N,N-dicyclohexylmethylamine and N,N-dimethylcyclohexylamine iminium products to yield formaldehyde and protonated N,N-dicyclohexylamine and N-methylcyclohexylamine, respectively. N-cyclohexylamine was obtained as the oxidation product of N,N-dicyclohexylamine [17]. 

The experimental results were rationalised by considering that planarity at the iminium intermediate (sp^2^-hybridised carbon) is required (Equation (5)), which will be favoured in the methyl group rather than in the cyclohexyl functional group. Moreover, steric effects contribute to the difficulty in accessing the α-carbon hydrogens in highly substituted tertiary amines (e.g., tri-isopropylamine and 9-*t*-butylazabicyclo[3.3.1]nonane) showing electrochemical reversible oxidation processes [19,35,36]. Similar reversibility is also observed with amines containing no hydrogens on the α-carbons [35,36]. Interestingly, the reversibility of 9-*t*-butylazabicyclo[3.3.1]nonane is partially lost when the *t*-butyl group is replaced by *i*-propyl [36].

Despite the well-known mechanism, research on adding nucleophiles to **2** is limited. Table 2 summarises the anodic oxidation of aliphatic and alicyclic amines in the presence of different nucleophiles. 

**Table 2:** Oxidation of tertiary amines in the presence of different nucleophiles.

Entry #	Starting Amine	Nucleophile	Product	% Yield	Ref.
1	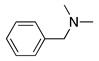	CH_3_OH	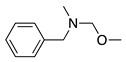	48	[37]
			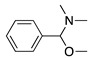	12	
2	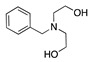	Intramolecular OH	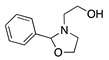	25	[37]
			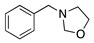	45	
3	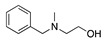	Intramolecular OH	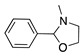	25	
			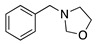	45	
4	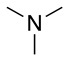	–CN	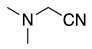	53	[38]
		DEM–	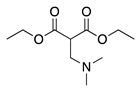	80	[39]
		DEP–	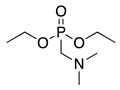	70	
5	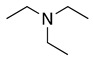	–CN	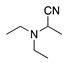	36	[38]
6	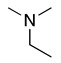	–CN	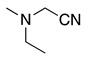	31	[38]
			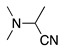		
7	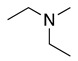	–CN	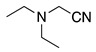	32	[38]
			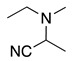		
8	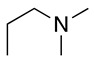	–CN	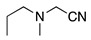	40	[38]
			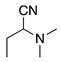		
9	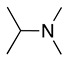	–CN	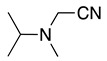	40	[38]
10	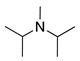	–CN	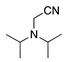	43	[38]
11	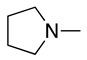	–CN	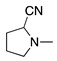	46	[38]
			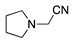		
12	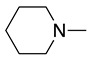	–CN	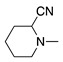	57	[38]
			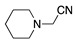		
13	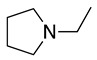	–CN	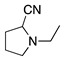	57	[38]
			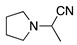		
14	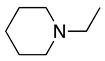	–CN	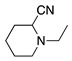	61	[38]
			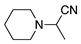		
15	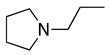	–CN	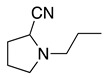	59	[38]
16	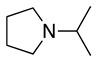	–CN	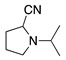	57	[38]
17	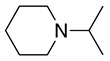	–CN	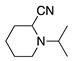	62	[38]
18	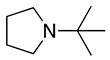	–CN	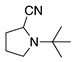	63	[38]
19	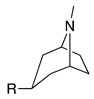	–CN	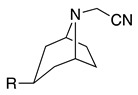	–	[40]
20	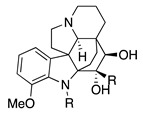	Intramolecular OH	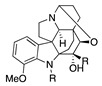	72	[41,42]
		–CH_2_CN	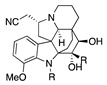	22	[42]
		–CN	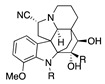	23	
21	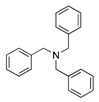	DEM–	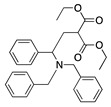	76	[39]
		DEP–	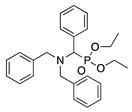	60	
22	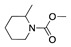	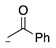	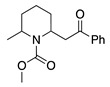	74	[43]
23	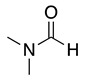	HCOO^−^	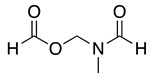	79	[44]
24	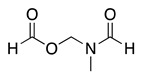	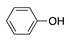	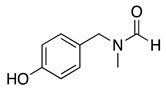	90	[44]
		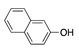	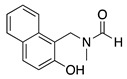	82	[44]
25	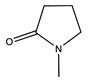	H_2_O	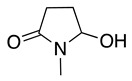	55	[45]
26	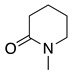	H_2_O	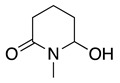	64	[45]
27	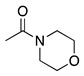	–CN	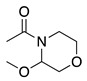	70	[46]
28	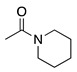	–CN	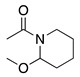	70	[46]
29	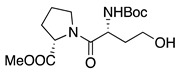	Intramolecular OH	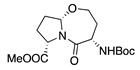	48	[47]
30	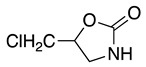	CH_3_OH	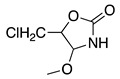	76	[48]
31	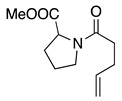	Intramolecular C=C	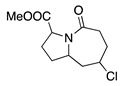	89	[49,50]
32	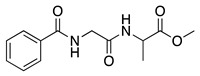	Intramolecular N	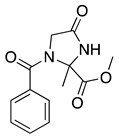	79	[51]
33	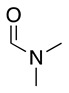	CH_3_OH	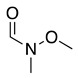	88	[52]
		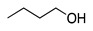	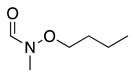	87	
		CH_3_COOH	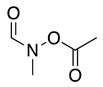	54	
34	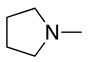	TsN_3_	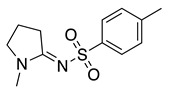	68	[53]
35	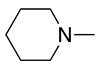	TsN_3_	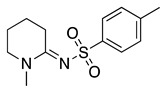	76	[53]
36	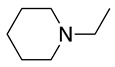	TsN_3_	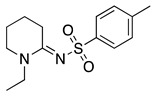	52	[53]

Abbreviations: –CN = cyanide ion; DEP– = diethyl phosphonate ion, DEM– = diethyl malonate ion, TsN_3_ = tosyl azide.

Aziridines are extremely reactive cyclic secondary amines that undergo ring-opening reactions in the presence of nucleophiles. They are used in textile chemicals, adhesives, binders, petroleum refining chemicals, fuels, lubricants, hardeners, etc. [54]. Aziridines undergo ring cleavage by anodic oxidation. For example, the electrochemical oxidation of 2-phenyl-2-ethylaziridine was studied in anhydrous methanol at 0 °C under a nitrogen atmosphere using a platinum working electrode [55]. The electrolysis showed a four-electron process with the production of (1,1-dimethoxypropyl)benzene in 50% yield. The reaction was postulated to proceed via initial two-electron oxidation to form an azaallyl cation intermediate (**10**, Equation (13)), which reacts with methanol to produce the imine **11**. This imine can suffer further two-electron oxidation to make **12** (12% yield, Equation (14)) and react with water to form propiophenone (observed in 6% yield) or react again with methanol to produce (1,1-dimethoxypropyl)benzene [55].

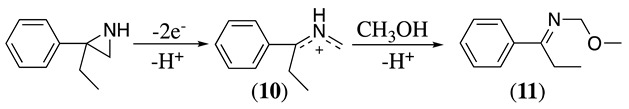
(13)

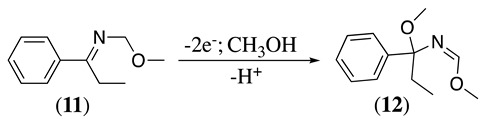
(14)

### 2.1. Catalysed Oxidation of Amines 

To decrease the overpotential needed for the direct oxidation of amines, as well as minimise the electrode surface fouling effect, increase the sensitivity, and enhance the reliability and reproducibility of the data, the catalytic oxidation of aliphatic amines with reversible redox couples acting as a mediator has been introduced. As discussed by Torriero et al., an ideal redox catalyst (or mediator) needs to have a standard reversible potential less positive than the oxidation potential of the substrate, exhibit fast electron-transfer kinetics, and be stable in both the oxidised and reduced form toward the species present in the reaction media [17,18,56,57]. Effective mediators that meet these requirements are based on ruthenium complexes, quinone, ferrocene (Fc), and their derivatives, either homogeneously dispersed in the solution or immobilised in a monolayer or multilayer configuration onto the electrode surface [17,18,58,59,60,61]. However, other options were equally reported. For example, the anodic oxidation of primary amines at nickel hydroxide electrodes in alkaline solutions forms nickel oxide hydroxide at a potential of 0.39 V vs. SCE, which reacts with propylamine and butylamine, forming propionitrile (84% yield) and butyronitrile (85% yield), respectively [62]. Nevertheless, when *i*-propylamine was used, acetone was formed in an 80% yield (Equation (15)).

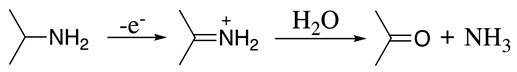
(15)

Chloride, bromide, and iodide have been used as redox catalysts (Table 3). For example, sodium chloride and sodium bromide were used as redox catalysts in a CH_3_CN-saturated NaCl aqueous (pH 4) solvent mixture using platinum as the working electrode for the electrochemical oxidation of cyclic aziridines to form keto nitriles in an 80% yield [63]. Keto nitriles are valuable intermediates for various synthetic transformations [64]. This reaction was explained by the generation of an azaallenyl intermediate cation, which is hydrated to form an α-hydroxyimine. Second oxidation by Cl^+^ followed by a rearrangement leads to the final keto nitrile (**13**, Equation (16)) [63].

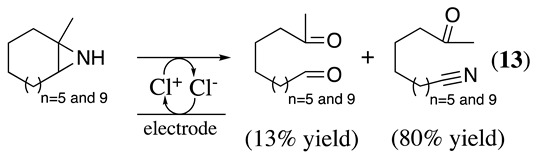
(16)

**Table 3:** Oxidation of amines using halides as redox catalysts.

Entry #	Starting Amine	Second Reactant	Redox Catalyst	Product	% Yield	Ref.
1	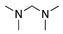	H_2_O	I^−^	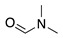	75	[65]
2	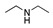	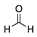	I^−^	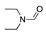	85	[65]
3	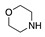	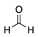	I^−^	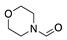	83	[65]
4	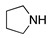	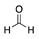	I^−^	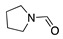	75	[65]
		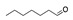	I^−^	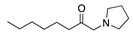	17	[65]
		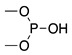	I^−^	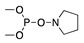	79	[66]
5	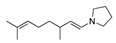	H_2_O	I^−^	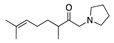	53	[65]
6	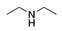	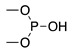	I^−^	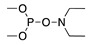	81	[66]
7	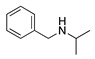	–	I^−^	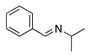	74	[67]
8	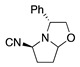	H_2_O	Br^−^	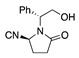	50	[68]
9	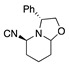	H_2_O	Br^−^	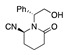	85	[68]
10	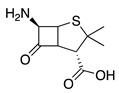	PhS-SPh	Br^−^	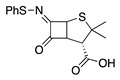	72	[69]
11	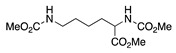	CH_3_OH	Cl^−^	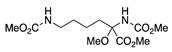	83	[70]
12	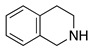	–	Cl^−^	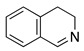	66	[67]
13	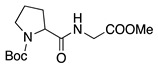	CH_3_OH	Cl^−^	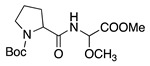	38	[51]
14	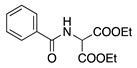	CH_3_OH	Cl^−^	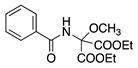	65	[71]

The previously postulated aziridine oxidation mechanism (Equations (13) and (14)) was also reported using different catalysts. For example, the acid-catalysed oxidation of N-benzylaziridine in methanol was reported to form 1,4,7,10-tetraphenyl-1,4,7,10-tetraazacyclododecane in 60% yield [72,73]. The same reaction catalysed by tris(4-bromophenyl)amine was also reported [4]. However, 1,4-dibenzylpiperazine was described as the product when the reaction was catalysed by iron porphyrin in a 5:1 CH_3_CN/H_2_O deaerated solvent system [74].

The two-electron oxidation of (3-amino-2,4-dihydroxyphenyl)(phenyl)methanone (**14**) to the corresponding benzoquinoneimine in the presence of different amines was studied in methanol containing either LiClO_4_, [Et_4_N][PF_6_], or [Et_4_N]ClO_4_ as the supporting electrolyte and using platinum as the working electrode (Equation (17)) [59,75]. The oxidation peak of the catalyst was observed at a potential of 0 V vs. SCE under these conditions.

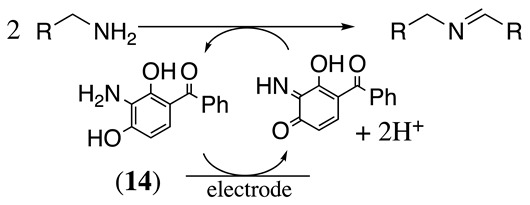
(17)

The amine transfers one electron per molecule to the benzoquinoneimine to regenerate the catalyst and afford the imine dimeric product. It was reported that only primary alkyl amines react with this mediator, with linear alkyl groups more reactive than branched ones, such as cyclohexyl or *t*-butyl groups. Meanwhile, phenylethylamine deactivates the catalyst following the reaction shown in Equation (18). It involves the reaction of the enamine form of the product (see Equation (5)) with the benzoquinoneimine form of the mediator following a Diels–Alder-type mechanism. The product **15** is unstable. Nevertheless, its two-electron oxidation product was isolated in a 65% yield [59].

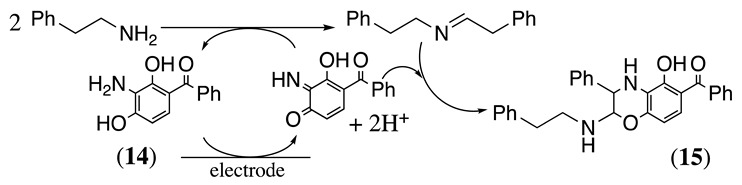
(18)

The reversible one-electron oxidation of para-substituted triphenylamines was also used as redox catalysts in acetonitrile and sometimes even in nucleophilic media for several amine oxidation reactions. The formal potential for the oxidation of the *p*-substituted triphenylamines depends on the electron-donative or electron-withdrawing nature of the *p*-substituent, covering the potential range of 0.6 to 1.8 V vs. SCE [25,76,77,78]. For example, the electrochemical oxidation of N-ethyl-*S*-(2-nitrophenyl)thiohydroxylamine was performed at 0.69 V vs. SCE in dichloromethane containing 0.1 M [Bu_4_N][ClO_4_] as the supporting electrolyte in the presence of *N*^1^,*N*^1^,*N*^4^,*N*^4^-tetrakis(4-bromophenyl)benzene-1,4-diamine as the catalyst (Equation (19)) [79]. The respective sulfenimine product was isolated in a 72% yield. Similarly, the electrochemical oxidation of benzylamine was performed at 0.99 V vs. SCE in acetonitrile containing 0.1 M [Bu_4_N][BF_4_] as the supporting electrolyte in the presence of tris(4-bromophenyl)amine as the catalyst (Equation (20)). Under these conditions, the respective iminium product was identified. Nevertheless, when the reaction was performed in a 50:50 dichloromethane:methanol solvent mixture, N-benzyl-1-phenylmethanimine was isolated in a 78% yield [80].

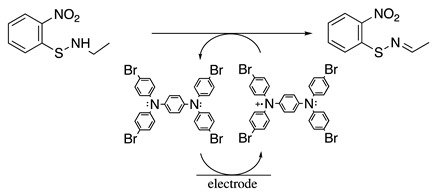
(19)

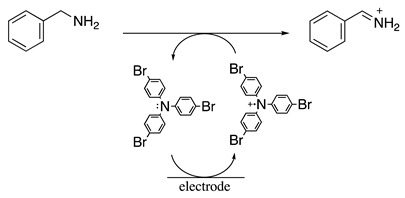
(20)

Pentaammineaquaruthenium(II) trifluoroacetate was used to catalyse the formation of amide functional groups from nitrile [81,82]. The reaction starts with the nitrile substituting the aqua ligand in the ruthenium complex (Equation (21)). Oxidation of this ruthenium(II) complex to ruthenium(III) permits the hydrolysis of nitrile to the amide. The reduction of this complex ensures the formation of the amide in a 70% yield and the recovery of the catalyst (Equation (22)) [82].

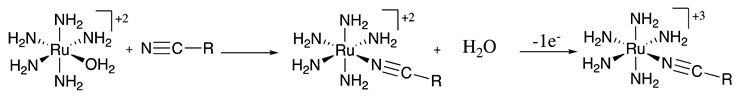
(21)

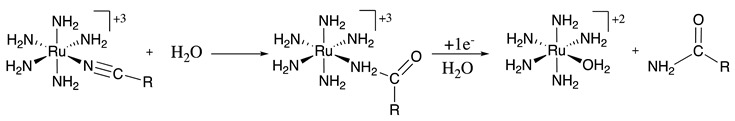
(22)

When a primary amine is used instead of a nitrile, the oxidation reaction forms the imine product or even the nitrile. For example, in the case of benzylamine, the final product was benzonitrile, with an 85% yield [82].

The oxidation of Fc to the corresponding ferrocenium cation, in the presence of different cyclohexylamines and n-alkylamines, was also studied in acetonitrile and dichloromethane containing 0.1 M [Bu_4_N][PF_6_] as the supporting electrolyte [17,18]. The reaction follows the mechanism described in Equations (1)–(7), obtaining the dealkylated amine as the main product (see above).

### 2.2. Examples of Applications

The electrochemical oxidation of cardiovascular-active kopsingine alkaloid in a CH_2_Cl_2_/CH_3_CN solvent system and in the presence of the non-nucleophilic base 2,6-lutidine results in an intramolecular cyclisation, generated by the attack of a hydroxy group to the electrochemically generated iminium ion, forming kopsidine A in 72% yield (Table 2, entry 20) [41,42]. 

Catharanthus alkaloids are valuable reagents for treating several cancers [83,84]. The electrochemical oxidation of the catharanthus roseus alkaloids catharanthine produces the iminium ion, which fragmented to give the highly cytostatic anhydrovinblastine. In the presence of methanol, the methoxy group is introduced in C16, making 16-methoxycleavamine in a 95% yield [85].

The 2,2,6,6-tetramethylpiperidine-1-oxyl (TEMPO) catalyst was used in the electrochemical N-demethylation of opiates resulting in noropiates, a critical intermediate in the opiate medicine partial chemical synthesis with a good yield of up to 83% [86]. This N-demethylation process follows through the anodic oxidation of TEMPO, resulting in an oxoammonium species, which then oxidises opiate to an iminium cation. Finally, this intermediate hydrolyses to yield noropiate.

The microsomal cytochrome P-450 monooxygenase system catalyses the dealkylation of secondary and tertiary amines and amides via an oxidation pathway. Therefore, the product of the enzymatic processes was compared with those discussed above to gain insight into the cytochrome P-450 catalysed N-dealkylation [87]. An agreement on the selectivity of dealkylation between microsomal and anodic dealkylations was observed [87].

Our group used the ferrocene-mediated oxidation of alkylamines to construct a selective and sensitive electrochemical biosensor to detect DNA hybridisation by employing the electrocatalytic activity of Fc-bearing Zn-cyclen complexes [60]. A sandwich-type approach was created, which involves hybridising a target probe with the immobilised thiolated capture probe attached to a gold electrode. Electrochemical signals are generated by voltammetric interrogation of Fc complexes that selectively and quantitatively bind to the duplex layers through strong chelation between the Zn-cyclen complexes and thymine bases within the DNA sequence. Coupling the redox chemistry of the surface-bound Fc-bearing Zn-cyclen complex and dimethylamine provides an electrocatalytic pathway that increases the sensitivity of the assay and allows the target DNA sequence to be detected at a 100 fM concentration level [60]. 

The selective electrochemical oxidation of tropane alkaloids to their nortropane derivatives using GC as the working electrode in a 2:1 ethanol:water or 2:1 methanol:water solvent system (0.1 M NaClO_4_) was described [40]. These oxidation products are important intermediates in the production of anticholinergics ipratropium and oxitropium bromide drugs. The reaction proceeds at room temperature following a mechanism similar to that described above to form the iminium intermediate (see Equations (1)–(5)), which reacts in the presence of water to form the respective nortropane (**16**) and formaldehyde (Equation (23)). When the oxidation reaction was performed in the presence of cyanide or **16** as nucleophiles, the respective addition reactions were observed (Table 2, entry 19 and Equation (24)) [7,40,88].

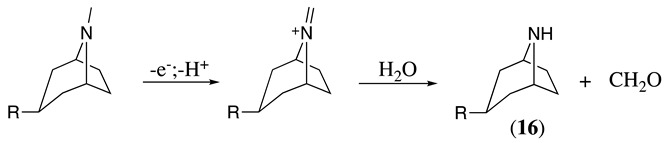
(23)

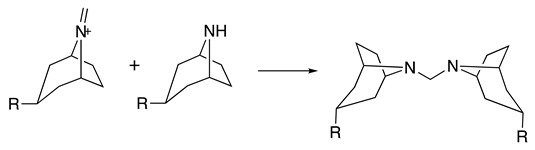
(24)

### 2.3. Alkanolamines, Amides, Carbamates and Lactams 

The term alkanolamines describes molecules that simultaneously contain amino and hydroxyl functional groups. The oxidation of these molecules in alkaline or neutral aqueous systems undergoes the previously mentioned dealkylation mechanism. For example, the oxidation of 2-(dimethylamino)-1-phenylethan-1-ol (**17**) yields benzaldehyde and the respective radical (Equation (25)), which disproportionate or loses a second electron to form an iminium intermediate (Equation (26)). The formation of formaldehyde and dimethylamine is observed after the interaction of this reactive intermediate with water molecules [89]. The electrochemical oxidation of ephedrine (2-(methylamino)-1-phenylpropan-1-ol) was evaluated using GCE as the working electrode in a pH 10 aqueous solution. The oxidation products benzaldehyde, acetaldehyde, and N-methylamine were obtained in yields of 65%, 68%, and 87%, respectively [90]. A similar mechanism and product distribution were observed for related alkanolamines, confirming the proposed mechanism [79,91,92].

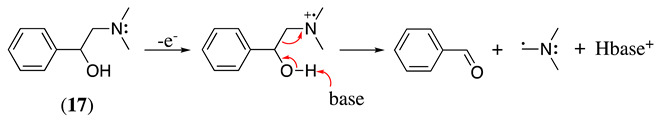
(25)

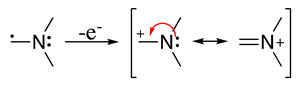
(26)

The electrochemical oxidation of 2,2′-(benzylazanediyl)bis(ethan-1-ol) was studied in alkaline methanol using a platinum working electrode. The major electrolysis products were 2-(2-phenyloxazolidin-3-yl)ethan-1-ol and 3-benzyloxazolidine in a 25% and 45% yield, respectively (Table 2, entry 2) [37]. Similarly, the anodic oxidation of 2-(benzyl(methyl)amino)ethan-1-ol under the same conditions leads to the formation of a mixture of 3-methyl-2-phenyl-oxazolidine and 3-benzyloxazolidine (Table 2, entry 3) [37].

The anodic oxidation of amides and carbamates is of considerable value from a synthetic point of view due to the stability of the intermediates [7]. Examples of synthetic applications can be observed in the alkaloids, enantiopure amino acids, chiral α-hydroxyl amide metabolites, and peptidomimetics areas [7,43,44,46,47,48,49,50,51,52,88,93,94,95,96,97,98,99,100,101,102,103,104,105,106,107,108,109]. The reaction proceeds via the initial one-electron oxidation of the nitrogen atom to the respective radical cation, which follows a similar pathway to that described before (Equations (1)–(5)) to produce the respective iminium intermediate (**18**, Equation (27)) [11,12]. The subsequent reactions of the iminium cations with nucleophiles (Equation (28)) have been extensively reported as amidoalkylation (examples in Table 2, entries 22–33). Typical nucleophiles include hydroxyl, enamines, isocyanides, enol esters, electron-rich olefins and aromatics, enol ethers, trimethylsilyl cyanide, vinyl and allyl silanes, and trialkylphosphites [44,46,47,48,49,50,51,52,93,94,95,96,97,98,99,100,101,102,103,104,105,106,107,108,110,111]. Meanwhile, as the iminium is in equilibrium with its enamine form (Equation (5)), this last intermediate can react with electrophilic groups (Equation (29)). Typical electrophiles include acyl chlorides and alkyl halides [112,113,114,115,116,117].

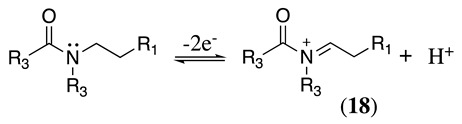
(27)

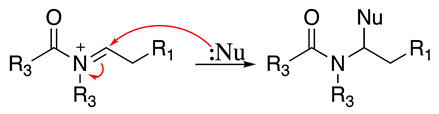
(28)

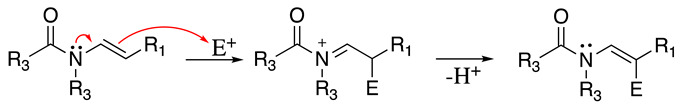
(29)

The conversion of an α-aminomalonic half-ester or an N-acylated amino acid via the Hofer–Moest reaction also allows the formation of **18** (Equation (30)). The reaction mechanism is similar to that shown above for alkanolamines (Equations (25) and (26)) and comprises an electrolytic decarboxylation reaction in neutral or alkaline solutions [118]. Because of the similarity in the mechanism with alkanolamines, the electrochemical oxidation of N-acylated β-amino alcohols will also generate the same N-acyliminium ion **18** (Equation (31)).

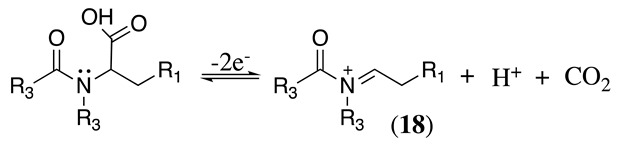
(30)

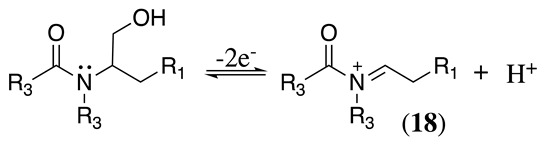
(31)

Secondary amide anions can be oxidised at potentials about 1 V more cathodic to the related amine (Table 1). The electrochemical irreversible one-electron oxidation produces the aminyl radical, which dimerises to make the respective hydrazine (Equation (32)). A similar result was obtained when secondary amines were electrochemically oxidised in an alkaline media [119,120,121]. Furthermore, it was observed that the reaction is effective if the intramolecular coupling occurs, producing cyclic hydrazines [119,120]. When di-n-butylamide is electrochemically oxidised in THF in the presence of di-n-butylamine, N,N-dibutyltetrahydrofuran-2-amine was formed in 30% yields [122].

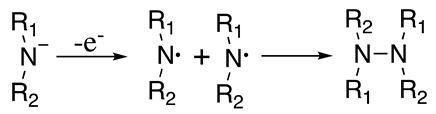
(32)

The electrochemical oxidation of lactams follows the same mechanism postulated for amides. Nevertheless, the oxidation position depends on the lactam ring size, structure of the N-alkyl substituent and electrolysis conditions. In the case of five- and six-member N-alkyl lactam rings, the oxidation and nucleophilic addition occur selectively at the lactam α-carbon to nitrogen (Table 2, entries 25, 26). Meanwhile, seven-member lactam rings show the reaction at the N-alkyl α-carbon [123,124,125,126]. Methoxylation at both positions was observed after the anodic oxidation of N-alkyl-β-lactam and N-benzyl-β-lactams in methanol using a platinum working electrode [45,127]. 

The anodic oxidation of 4-carboxy-2-azetidinone (**19**) in acetonitrile follows the decarboxylation mechanism described in Equation (30), which in the presence of sodium acetate produces 4-acetoxy-2-azetidinones (**20**) in 76% yield (Equation (33)) [128].

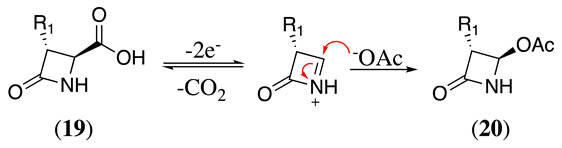
(33)

## 3. Aniline and Derivatives

The electrochemical anodic oxidation of aromatic amines has been studied extensively. The prime focus of all those investigations was to establish the oxidation mechanism under multiple electrolysis setups due to their wide variety of applications in synthetic and polymer chemistry and pharmaceutical and dye industries. In the literature, several reviews of the electrochemical oxidation of aromatic amines are available [4,7,129]. Hence, the anodic oxidation mechanism of aniline and its derivatives, which are not comprehensively discussed in the available literature, is addressed in this work.

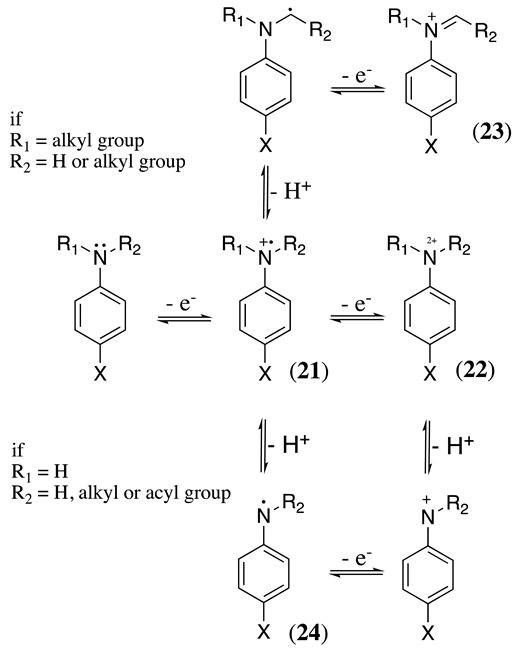
(34)

The electrochemical potential at which aniline derivatives oxidise depends on the nature of the substituents present both at the nitrogen and the aromatic ring. Electron-withdrawing substituents shift the oxidation redox potential towards more positive values, while the electron-donating substituents change the oxidation potential in the opposite direction (Table 1). Nevertheless, a common feature in all cases is that the anodic oxidation of aniline and its derivatives starts with the nitrogen loss of one electron to yield the radical cation **21** (Equation (34)). After this point, the oxidation mechanism depends on the substitution level at the nitrogen atom and the basicity of the reaction media. For N,N-disubstituted anilines under basic conditions, the oxidation mechanism is similar to that previously described for aliphatic amines with the formation of the respective iminium product **23** (Equation (34)). However, nitrogen deprotonation reaction is predominantly observed in mono- or no N-substituted anilines, producing the respective radical intermediate **24**. Alternatively, the subsequent oxidation produces **22**, which may deprotonate to generate the respective nitrenium cation (Equation (34)) [130]. 

Writing the resonance structures for **24** may help to understand all the reaction pathways discussed below. Based on this resonance, it is possible to see that the radical may reside on the nitrogen atom or delocalised at the *ortho* (**25**) and *para* (**26**) positions in the aromatic ring (Equation (35)).

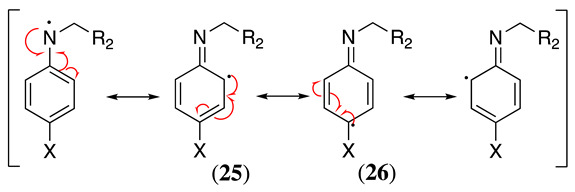
(35)

Therefore, it is evident that a diverse range of products can be formed, where two radical cations, two radicals, a radical cation and a radical, or a cation and a starting molecule can couple together via the formation of a new C-C, C-N, or N-N bond [131]. For example, the following general reactions can be postulated [132]:

(i). Two resonance structures **26** can react through a tail-to-tail coupling to form dimer **27** (Equation (36)).
(ii). The nitrenium cation can react with starting molecule through a head-to-tail coupling to form dimer **28**. The same outcome is obtained by reacting **24** with **26** (Equation (37)).
(iii). Two resonance structures **24** can react via a head-to-head coupling to form dimer **30** (Equation (38)).

In the case of N,N-disubstituted anilines under basic conditions, iminium product **23** can react with nucleophiles present in the solution to produce the respective addition (or an α-substitution if we consider the starting molecule) products (Equation (39)).

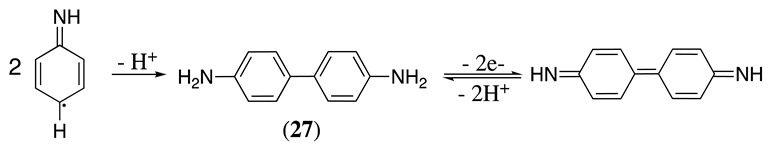
(36)

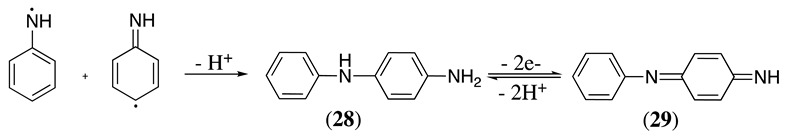
(37)

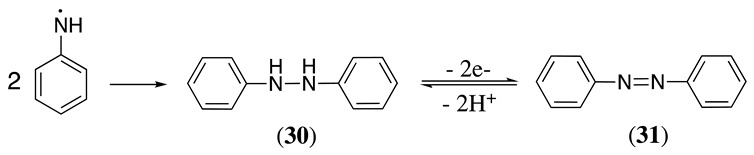
(38)

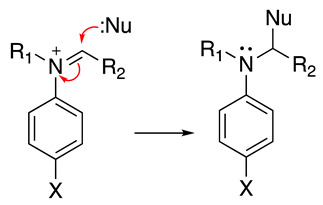
(39)

### 3.1. Aniline and Para-Substituted Anilines

The electrochemical anodic oxidation of aniline may follow the different mechanisms outlined in Equations (36)–(38). The major product obtained depends on the aqueous or nonaqueous, acidic, basic, or neutral pH media conditions. Acidic conditions, which are usually used to facilitate the dissolution in aqueous media of otherwise insoluble aniline derivatives, may require the addition to the reaction mechanism of an initial deprotonation homogeneous chemical reaction before the first electron transfer, which most of the time is not taken into consideration when reporting the oxidation mechanisms under these conditions.

The three different anodic oxidation products of aniline are as follows. When there is no *para* substituent (or X=H), the intermediate **26** can undergo tail-to-tail coupling with itself or head-to-tail coupling with **24** in an acidic aqueous medium, producing benzidine (**27**) in a small yield and *p*-aminodiphenylamine (**28**), respectively (Equations (36) and (37)), both of which can be reversibly oxidised via a two-electron and two-proton process [133]. The head-to-head dimerisation reaction to form hydrazobenzene **30** was reported both in acidic and basic reaction conditions [23,134]. In the case of **28**, the oxidation product may undergo slow acid-catalysed hydrolysis to produce *p*-benzoquinone and the parent amine, in this case, aniline (Equation (40)). The anodic oxidation of aniline in a nonaqueous medium (e.g., acetonitrile) generates **28** as the main product, which is stable under these conditions due to the non-availability of protons to catalyse the previously mentioned hydrolysis.

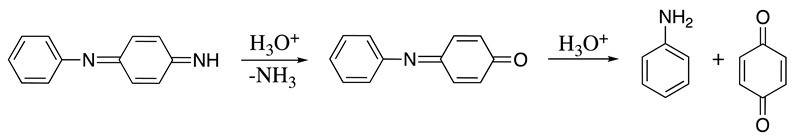
(40)

When there is a substituent at *para* position (X = OCH_3_, OC_2_H_5_, Cl, CH_3_, COOH, NO_2_), intermediate **24** undergoes a head-to-head coupling predominantly to produce hydrazobenzene **30** derivatives [133]. 

When aniline and its derivatives are oxidised using an acetonitrile/pyridine solvent system, azobenzene **31** is produced in 39% yield, with **30** appearing as a by-product [23]. The head-to-head coupling of radical cations in the presence of pyridine can be explained using Equations (34) and (38), where the radical cation **21** reacts with pyridine in a Bronsted–Lowry acid–base reaction producing **24** (in this case, R_1_ = R_2_ = H), which undergoes head-to-head coupling to yield **30** (Equation (38)). A simple explanation for the generation of **29** could be the head-to-tail coupling of the neutral radical and the subsequent two-electron and two-proton oxidation process. Pyridine plays the crucial role of proton acceptor in this reaction. For example, electrochemical oxidation of *p*-nitroaniline and *p*-chloroaniline in acetonitrile/pyridine produced 4,4′-dinitroazobenzene (39.2% yield) and 4,4′-dichloroazobenzene (24.3% yield), respectively [23]. The anodic oxidation of 2,4-dinitroaniline produced 2,2′,4,4′-tetranitroazobenzene in a 38% and 31% yield when a 3:5 water:acetonitrile and 1:3 water:DMF solvent mixtures were used, respectively [135]. Aniline was oxidised in 1M KOH, forming hydrazobenzene, which produced **31** (Equation (38)) in a 30% yield after further oxidation [136]. 

### 3.2. N-Substituted Anilines

After the first oxidative electron transfer step, the mono N-alkyl or N-aryl substituted anilines show a relatively more stable radial cation due to the stabilisation effect of its electron-donating substituents. There are four possible ways this radical cation undergoes subsequent reactions depending on the reaction conditions. The first three pathways are identical to those explained for aniline (Equations (36)–(38)). The fourth one is in the presence of nucleophiles following the nucleophilic substitution at the *α*-carbon to the nitrogen through an iminium ion intermediate (Equation (39)). For example, if the electrolysis reaction is performed under high current density conditions (e.g., 4 mA cm^2^), the high concentration of radical cations produced and the lower concentration of parent molecules remaining at the electrode surface leads to a tail-to-tail coupling product. However, when the reactions are performed under strongly basic conditions and at lower current densities (e.g., ≤0.8 mA cm^2^), the higher concentration of parent molecules at the electrode surface favours the head-to-tail coupling product formation, which in this case may undergo oxidation and hydrolysis to yield *p*-benzoquinone and starting amine [7]. Moreover, the bulkiness of the N-alkyl group plays a crucial role in product formation. For example, in acidic conditions, electrolysis of N-methylaniline at a high current density produces *p*-benzoquinone (50%) and N,N′-dimethylbenzidine (50%); N-ethylaniline produces *p*-benzoquinone (40%) and N,N′-diethylbenzidine (60%); N-*t*-butylaniline in acetonitrile at a high current density yields N,N′-di-*t*-butylbenzidine (100%) [133].

The oxidation of diphenylamine in acetonitrile using platinum electrodes generates the electroactive N,N′-diphenylbenzidine product [137]. The same type of coupling can be observed even when the oxidation of diphenylamine is carried out under weakly basic conditions [138]. Nevertheless, under weakly basic conditions, if a methoxy group is present in the *para* position, as in the case of dianisylamine, 2,7-dimethoxy-9,10-dianisyl-9,10-dihydrophenazine is formed as the main product [138]. 

N-alkyl substituted anilines also undergo nucleophilic substitution at the α-carbon to the nitrogen (Equation (39)) in the presence of nucleophiles such as enol ethers [139]. For example, when N-methylaniline is oxidised under constant current in methanol containing LiClO_4_ as the supporting electrolyte and in the presence of 2,3-dihydrofuran, the tetrahydroquinoline **32** (12%) and the acetal **33** (6.3% *trans*-isomer and 5% *cis*-isomer) derivatives were obtained (Equation (41)) [139].

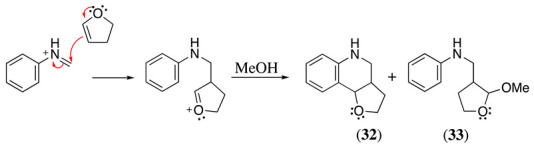
(41)

The anodic oxidation of diphenylamine and its derivatives, *o*-methoxydiphenylamine, *o*-methyldiphenylamine, and *m*-methyldiphenylamine in methanol containing sodium cyanide as the supporting electrolyte and the source of nucleophile was performed, resulting in the formation of the respective cyanodiphenylamines in relatively good yield [140]. For example, the cyanation of diphenylamine happened at the *para* position of both phenyl rings to produce bis(4-cyanophenyl)amine in a 61% yield (Equation (42)). However, in the case of *o*-methoxydiphenylamine, *o*-methyldiphenylamine, and *m*-methyldiphenylamine, the nucleophilic aromatic substitution happened only at the *para* position of the substituted phenyl ring in a 40–50% yield.

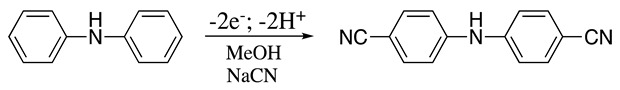
(42)

### 3.3. N,N-Disubstituted Anilines

The N,N-dialkylanilines and N,N-diarylanilines undergo a similar oxidation mechanism as discussed earlier for N-substituted anilines. For example, in the absence of *para* substituent, the tail-to-tail coupling producing derivatives of **27** was observed. The resulting dimer is more easily oxidised than the starting material, generating the respective quinoidal diimino cation [131].

The anodic oxidation of N,N-dimethylaniline derivatives has been extensively studied in different reaction media and it was found that the primary product formed through tail-to-tail coupling is N,N,N′,N′-tetramethylbenzidiene [24,141,142,143,144]. However, if the electrochemical oxidation of N,N-dimethylaniline is performed in the presence of phenothiazine, a *para*-selective head-to-tail coupling happens with the formation of N,N,N′,N′-tetramethylbenzidiene as a secondary product (Equation (43)) [145]. Similarly, anodic oxidation of 8-aminoquinolines and *para*-substituted N,N-dimethylaniline in the presence of sodium sulfinates generates radical–radical cross-coupling, resulting in the formation of sulfones with a new C-S bond [146,147].

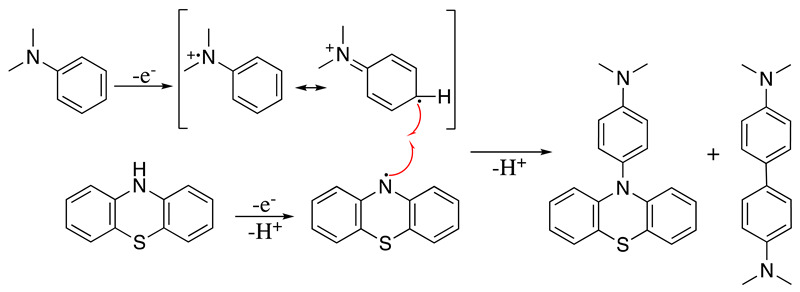
(43)

The oxidation of N,N-diphenylaniline in acetonitrile undertakes tail-to-tail coupling to yield tetraphenyl benzidine, and this dimer undergoes further oxidation, resulting in a quinoidal dication [25]. On the other hand, the oxidation of N,N-dimethyl-*p*-anisidine in acetonitrile containing traces of water results in the formation of 4-dimethylaminophenol [148]. The anodic oxidation of 4-dimethylaminophenol in aqueous media produces N,N-dimethylbenzoquinoneimine, which undergoes further hydrolysis to yield benzoquinone and dimethylamine in a reaction similar to that described in Equation (40) [149]. The oxidation of N,N-dimethyl-*p*-toluidine in acetonitrile leads to the tail-to-tail dimerisation product 4,4′-(ethane-1,2-diyl)bis(N,N-dimethylaniline) [148,150,151]. 

N,N-dialkylanilines can undergo nucleophilic substitution in α-position to nitrogen. For example, the anodic oxidation of N,N-dimethylaniline in methanol under basic conditions yields two products, *α*-methoxy-N-N-dimethylaniline and *α*-*α*′-dimethoxy-N-N-dimethylaniline in a ratio of 6:1 [37,152]. However, the methoxylation of N-ethyl-N-methylaniline predominately occurs at the methyl group, resulting in a highly regioselective reaction (Equation (44)) [153]. Similarly, the oxidation of N-ethyl-N-methylaniline in acetonitrile containing tetraethylammonium cyanide as the supporting electrolyte and the nucleophile source produced cyanation preferentially at the methyl position (64% yield) with the formation of 2-(methyl(phenyl)amino)propanenitrile by-product in a 34% yield [154].

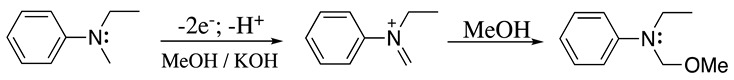
(44)

The anodic cyanation of 3,5-dimethyl-2,3,4,5-tetrahydro-1-benzazepine was performed at a carbon electrode in methanol containing sodium cyanide and lithium acetate as the supporting electrolyte. This reaction produced the *trans*-α-cyano derivative **34** and side product **35**, where the cyanation occurs at the side chain (Equation (45)) [155].

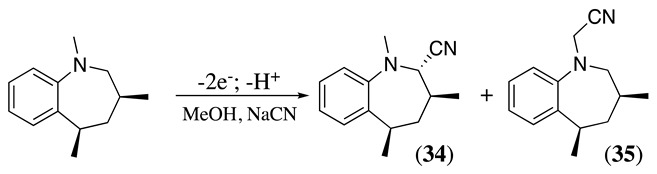
(45)

### 3.4. Aminophenols and N-Acylated Anilines 

Like other anilines, the aminophenol radical cation formed after the initial electron transfer may undergo dimerisation reactions, resulting in a new N-N, C-C, or C-N bond depending on the reaction condition. These dimerisation pathways can be explained using Equations (36)–(38). For example, the anodic two-electron oxidation of *p*-aminophenol to quinoneimine is a well-documented reaction, which may undergo hydrolysis in acidic media, resulting in the formation of the respective *p*-benzoquinone (Equation (46)) [156,157]. The oxidation of *o*-aminophenol in basic or neutral media produces a dimer formed through N-N coupling of *o*-aminophenol cation radicals similar to that reported in Equation (38) [158]. However, if the same reaction was performed in acidic solutions, the C-N coupling of *o*-aminophenol results in the formation of 2-aminophenoxazin-3-one [158].

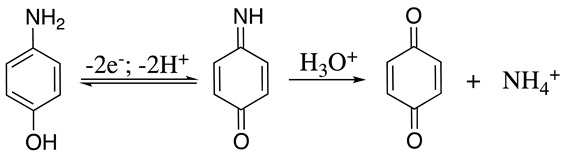
(46)

When the anodic oxidation of *o*- or *p*-aminophenol derivatives results in a stable quinoneimine intermediate, they can then react with nucleophiles present in the solution. For example, the anodic oxidation of 1-(4-(4-hydroxyphenyl)piperazin-1-yl)ethan-1-one, **36**, in a phosphate buffer/acetonitrile solvent mixture undergoes a two-electron and one proton transfer process to yield the respective quinoneimine, **37**, which, in the presence of 2-mercaptobenzothiazole, produces the *mono*-thiolated product **38** (Equation (47)) [159]. This product can undergo a second two-electron oxidation process and add a second 2-mercaptobenzothiazole molecule to the remaining *ortho* position to the OH-group, generating the respective *di*-thiolated product in a 93% yield.

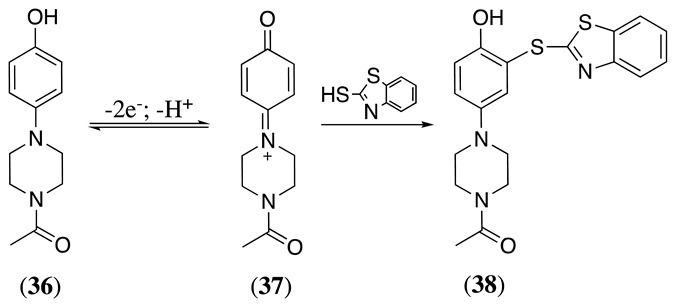
(47)

The anodic oxidation of N-acylated aniline derivatives can yield various products depending on the nature of the reactants and reaction conditions. The reaction proceeds, as discussed in Equation (34), with the formation of the radical **24**. Then, the radicals dimerise to create a new N-N or C-C bond. For example, the anodic oxidation of substituted anilides at graphite electrodes in acetonitrile containing potassium acetate and acetic acid produced N,N-diarylhydrazine derivatives, **39**, in a ca. 63% yield (Equation (48)) [160]. Meanwhile, when the reaction is performed at a glassy carbon electrode in methanol containing tributylmethylammonium methylsulfate as the supporting electrolyte and 1,1,1,3,3,3-hexafluoro-2-propanol (HFP) as a stabiliser, the C-C coupling product **40** is obtained in a 51% yield (Equation (49)) [161,162,163]. It was postulated that HFP may help to prolong the amidyl radical intermediate lifetime. Alternatively, the HFP alkoxide generated in situ at the cathode electrode may help during the initial electron-transfer process [161,162,163].

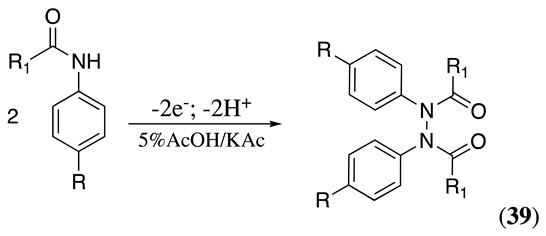
(48)

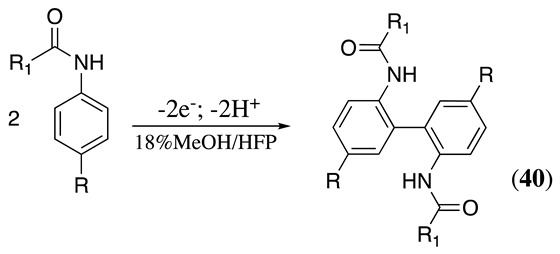
(49)

Like *o*- or *p*-aminophenol derivatives, quinoneimine intermediates also are produced upon the oxidation of amino-protected *o*- or *p*-aminophenol derivatives. These quinoneimine intermediates are valuable electrophiles to perform different organic reactions. N-(4-hydroxyphenyl)-4-methylbenzenesulfonamide can be electrochemically oxidised at a graphite electrode in acetate buffer/acetonitrile solvent mixture to produce **41** via a two-electron two-proton process (Equation (50)) [164]. This quinoneimine can react with sodium benzenesulfinate to produce N-[4-hydroxy-5-(phenylsulfonyl)phenyl]benzenesulfonamide, **42**, in a 55% yield. The presence of the amino-protecting group increases the regioselectivity of the reaction, happening exclusively at the *ortho*-position to the -OH group [164].

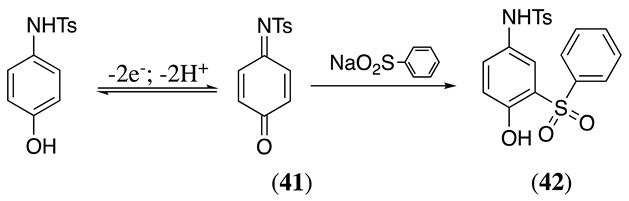
(50)

### 3.5. Catalysed Oxidation of Aniline and Its Derivatives

The electrocatalytic oxidation of N,N-dialkylaniline in acetonitrile containing water, lutidine (as the base), NaClO_4_ as the supporting electrolyte, and TEMPO as the catalyst was reported to produce N-alkylformanilide and N-alkylaniline as a secondary product [165,166]. The electrochemically generated oxoammonium cation (**43**, Equation (51)) reacts with N,N-dialkylaniline, generating the iminium derivative **44** (Equation (52)). As previously mentioned, the iminium can hydrolyse in the presence of water, resulting in the formation of N-alkylaniline, **45** via an amino alcohol intermediate. Alternatively, it can be further oxidised for a second mol of **43** to form N-alkylformanilide derivative, **46** (Equation (53)), in a 75–92% yield, depending on the nature of the R group [165].

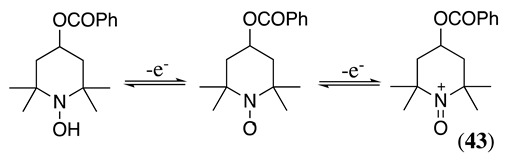
(51)

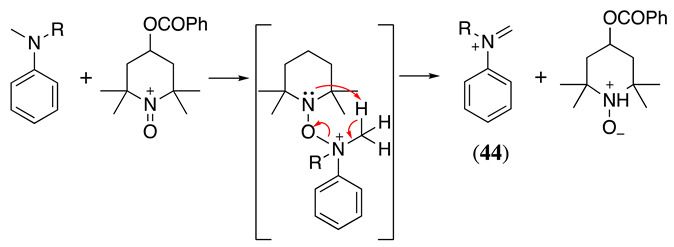
(52)

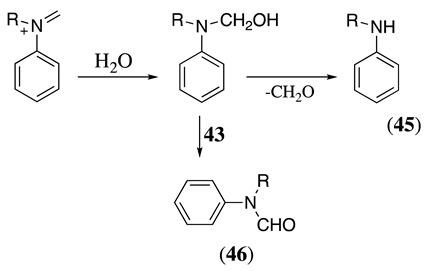
(53)

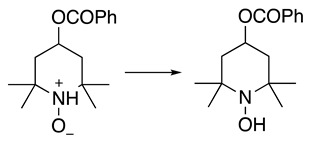
(54)

## 4. Conclusions

Over the past few decades, electrochemical anodic oxidation of amines has achieved remarkable advancement and shown great promise as a tool for organic transformations. The idea of creating radical precursors through electrode-initiated electron transfer provides a greener choice than conventional chemical reagents and can offer the possibility of using solvents in a sustainable form. In this work, we discussed the electrochemical oxidation mechanism of aliphatic amines, amides, aniline and aniline derivatives, carbamates, and lactams, either directly oxidised at different electrode surfaces or indirectly oxidised by a reversible redox molecule, in which the reactive form was generated in situ. A common feature in the oxidation mechanism of these amines is that the reaction starts with the nitrogen loss of one electron to yield a radical cation, which is stabilised by a following deprotonation step. This means that hydrogen at the α-carbon to the amine, directly connected to the amine, or at the aromatic ring becomes acidic in this process, facilitating α-substitution, radical–radical dimerisation, or nucleophilic aromatic substitution reaction, depending on the substituents present on the amine molecule under study and the experimental conditions. Meanwhile, if the formation of an iminium in equilibrium with its enamine form is possible, then this last intermediate can react with electrophilic groups, facilitating β-substitutions.

This work could help readers understand the comparative similarities and differences in the anodic oxidation mechanism of different amines.

## Figures and Tables

**Table 1:** Oxidation potential of different aliphatic and aromatic amines.

Amine	Solvent	Supporting Electrolyte	Working Electrode	*E^ox^* ^a^ (V vs. SCE)	Ref.
propylamine	CH_3_CN	0.1 M Na[ClO_4_]	Pt	1.38	[10]
butylamine	DMF	0.1 M [Bu_4_N][BF_4_]	GC	1.36	[16]
	EtOH	0.1 M LiClO_4_	GC	1.22	[20]
	THF	0.1 M LiClO_4_	GC	1.15	[21]
pentylamine	CH_3_CN	0.1 M Na[ClO_4_]	Pt	1.45	[10]
hexylamine	DMF	0.1 M [Bu_4_N][BF_4_]	GC	1.36	[16]
nonylamine	CH_3_CN	0.1 M Na[ClO_4_]	Pt	1.48	[10]
*t*-butylamine	DMF	0.1 M [Bu_4_N][BF_4_]	GC	1.44	[16]
	THF	0.1 M LiClO_4_	GC	1.21	[21]
	CH_3_CN	0.1 M Na[ClO_4_]	Pt	1.40	[10]
butylamide	THF	0.1 M LiClO_4_	GC	0.16	[21]
*t*-butylamide	THF	0.1 M LiClO_4_	GC	−0.10	[21]
cyclohexylamine	DMF	0.1 M [Bu_4_N][BF_4_]	GC	1.39	[16]
	THF	0.1 M LiClO_4_	GC	1.26	[21]
cyclohexylamide	THF	0.1 M LiClO_4_	GC	0.05	[21]
N-methylacetamide	CH_3_CN	0.2 M NaClO_4_	GC	1.81	[12]
N-acetylethylenediamine	EtOH	0.1 M LiClO_4_	GC	1.27	[20]
dopamine	EtOH	0.1 M LiClO_4_	GC	1.22	[20]
N-(5-aminopentyl)biotinamide	EtOH	0.1 M LiClO_4_	GC	1.22	[20]
diethylamine	CH_3_CN	0.1 M [Bu_4_N][PF_6_]	GC	1.10	[18]
dipropylamine	CH_3_CN	0.1 M NaClO_4_	Pt	1.00	[10]
dibutylamine	DMF	0.1 M [Bu_4_N][BF_4_]	GC	1.11	[16]
	THF	0.1 M LiClO_4_	GC	0.94	[21]
	CH_3_CN	0.1 M NaClO_4_	Pt	1.07	[10]
dibutylamide	THF	0.1 M LiClO_4_	GC	−0.12	[21]
dibenzylamine	CH_3_CN	0.1 M NaClO_4_	Pt	1.23	[10]
di-isopropylamine	DMF	0.1 M [Bu_4_N][BF_4_]	GC	1.15	[16]
di-isobutylamine	DMF	0.1 M [Bu_4_N][BF_4_]	GC	1.11	[16]
di-*sec*-butylamine	DMF	0.1 M [Bu_4_N][BF_4_]	GC	1.16	[16]
	CH_3_CN	0.1 M NaClO_4_	Pt	1.16	[10]
dipentylamine	CH_3_CN	0.1 M NaClO_4_	Pt	1.11	[10]
bis-2-ethylhexylamine	DMF	0.1 M [Bu_4_N][BF_4_]	GC	1.07	[16]
N-methylbutylamine	EtOH	0.1 M LiClO_4_	GC	1.00	[20]
N-ethylbutylamine	EtOH	0.1 M LiClO_4_	GC	0.99	[20]
dicyclohexylamine	DMF	0.1 M [Bu_4_N][BF_4_]	GC	1.06	[16]
	CH_3_CN	0.1 M [Bu_4_N][PF_6_]	GC	1.49	[17]
N,N-dimethylacetamide	CH_3_CN	0.2 M NaClO_4_	GC	1.32	[12]
trimethylamine	CH_3_CN	0.1 M NaClO_4_	Pt	1.05	[10]
triethylamine	EtOH	0.1 M LiClO_4_	GC	0.83	[20]
	DMF	0.1 M [Bu_4_N][BF_4_]	GC	0.94	[16]
	CH_3_CN	0.1 M [Bu_4_N][PF_6_]	GC	0.88	[18]
	CH_3_CN	0.1 M NaClO_4_	Pt	0.95	[10]
tripropylamine	DMF	0.1 M [Bu_4_N][BF_4_]	GC	0.95	[16]
	CH_3_CN	0.1 M NaClO_4_	Pt	0.93	[10]
tributylamine	DMF	0.1 M [Bu_4_N][BF_4_]	GC	0.88	[16]
	CH_3_CN	0.1 M NaClO_4_	Pt	0.78	[10]
tripentylamine	DMF	0.1 M [Bu_4_N][BF_4_]	GC	0.91	[16]
	CH_3_CN	0.1 M NaClO_4_	Pt	0.89	[10]
tribenzylamine	CH_3_CN	0.1 M NaClO_4_	Pt	0.99	[10]
tri-isopropylamine	THF	0.1 M [Bu_4_N]ClO_4_	GC	0.76	[19]
tri-isobutylamine	DMF	0.1 M [Bu_4_N][BF_4_]	GC	0.98	[16]
N,N-dicyclohexylmethylamine	CH_3_CN	0.1 M [Bu_4_N][PF_6_]	GC	1.04	[17]
N,N-dimethylcyclohexylamine	CH_3_CN	0.1 M [Bu_4_N][PF_6_]	GC	1.18	[17]
N,N-dimethylbutylamine	EtOH	0.1 M LiClO_4_	GC	0.99	[20]
4-nitrobenzylamine	DMF	0.1 M [Bu_4_N][BF_4_]	GC	1.42	[16]
	CH_3_CN	0.1 M [Bu_4_N][BF_4_]	GC	1.58	[16]
3-nitrobenzylamine	DMF	0.1 M [Bu_4_N][BF_4_]	GC	1.51	[16]
	CH_3_CN	0.1 M [Bu_4_N][BF_4_]	GC	1.78	[16]
N-methyl-3-nitrobenzylamine	DMF	0.1 M [Bu_4_N][BF_4_]	GC	1.25	[16]
	CH_3_CN	0.1 M [Bu_4_N][BF_4_]	GC	1.33	[16]
N,N-dimethyl-3-nitrobenzylamine	DMF	0.1 M [Bu_4_N][BF_4_]	GC	1.01	[16]
	CH_3_CN	0.1 M [Bu_4_N][BF_4_]	GC	1.07	[16]
pyrrolidine	CH_3_CN	0.1 M [Bu_4_N][PF_6_]	GC	1.16	[18]
pyrrole	CH_3_CN	0.5 M NaClO_4_	Pt	1.06 *	[22]
pyridine	CH_3_CN	0.5 M NaClO_4_	Pt	2.12 *	[22]
N,N-dipropylpropionamide	CH_3_CN	0.2 M NaClO_4_	GC	1.26	[12]
aniline	CH_3_CN	0.5 M NaClO_4_	Pt	0.90 *	[23]
*p*-nitroaniline	CH_3_CN	0.5 M NaClO_4_	Pt	1.39 *	[23]
*p*-bromoaniline	CH_3_CN	0.5 M NaClO_4_	Pt	0.97 *	[23]
*p*-chloroaniline	CH_3_CN	0.5 M NaClO_4_	Pt	0.96 *	[23]
*p*-anisidine	CH_3_CN	0.5 M NaClO_4_	Pt	0.62 *	[23]
*o*-anisidine	CH_3_CN	0.5 M NaClO_4_	Pt	0.70 *	[23]
diphenylamine	CH_3_CN	0.1 M NaClO_4_	Pt	0.83 *	[24]
triphenylamine	CH_3_CN	0.1 M [Et_4_N]ClO_4_	Pt	0.98	[25]
N,N-dimethylaniline	CH_3_CN	0.1 M [Bu_4_N][PF_6_]	Pt	0.76	[26]
N,N-diethylaniline	CH_3_CN	0.5 M NaClO_4_	Pt	0.70 *	[23]
N,N-diethyl-*p*-chloroaniline	CH_3_CN	0.5 M NaClO_4_	Pt	0.83 *	[23]
N,N-dimethyl-*p*-chloroaniline	CH_3_CN	0.5 M NaClO_4_	Pt	0.85 *	[23]
ethylphenylamine	CH_3_CN	0.5 M NaClO_4_	Pt	0.76 *	[23]
di-4-tolylamine	CH_3_CN	0.1 M NaClO_4_	Pt	0.70 *	[24]
N,N-tetramethylbenzidine	CH_3_CN	0.1 M [Pr_4_N]ClO_4_	Pt	0.43 *	[27]
1-dimethylaminonaphthalene	CH_3_CN	0.1 M [Pr_4_N]ClO_4_	Pt	0.75 *	[27]
2-dimethylaminonaphthalene	CH_3_CN	0.1 M [Pr_4_N]ClO_4_	Pt	0.67 *	[27]
azobenzene	CH_3_CN	0.5 M NaClO_4_	Pt	1.69 *	[23]
4,4-dichloroazobenzene	CH_3_CN	0.5 M NaClO_4_	Pt	1.80 *	[23]
4,4-dimethoxyazobenzene	CH_3_CN	0.5 M NaClO_4_	Pt	1.34 *	[23]
N,N,N′,N′-tetramethyl-*m*-phenylenediamine	CH_3_CN	0.1 M [Pr_4_N]ClO_4_	Pt	0.62 *	[28]
N,N,N′,N′-tetramethyl-*p*-phenylenediamine	CH_3_CN	0.1 M [Pr_4_N]ClO_4_	Pt	0.20 *	[28]
N,N-dimethyl-*m*-anisidine	CH_3_CN	0.1 M [Pr_4_N]ClO_4_	Pt	0.79 *	[28]
N,N-dimethyl-*p*-anisidine	CH_3_CN	0.1 M [Pr_4_N]ClO_4_	Pt	0.63 *	[28]
3,4-dimethoxy-N,N-dimethylaniline	CH_3_CN	0.1 M [Pr_4_N]ClO_4_	Pt	0.50 *	[28]
3,5-dimethoxy-N,N-dimethylaniline	CH_3_CN	0.1 M [Pr_4_N]ClO_4_	Pt	0.80 *	[28]
N,N,N′,N′-tetramethyl-*o*-phenylenediamine	CH_3_CN	0.1 M [Pr_4_N]ClO_4_	Pt	0.58 *	[28]
N,N-dimethyl-*o*-anisidine	CH_3_CN	0.1 M [Pr_4_N]ClO_4_	Pt	0.78 *	[28]
2,4-dimethoxy-N,N-dimethylaniline	CH_3_CN	0.1 M [Pr_4_N]ClO_4_	Pt	0.57 *	[28]

^a^ oxidation peak potential is reported, except those with (*), which are the *E*_1/2_. Abbreviations: DMF = dimethylformamide; CH_3_CN = acetonitrile; EtOH = ethanol; THF = tetrahydrofuran; [Bu_4_N] = tetrabutylammonium; [Et_4_N] = tetraethylammonium; [Pr_4_N] = tetrapropylammonium; [PF_6_] = hexafluorophosphate; [BF_4_] = tetrafluoroborate; SCE = saturated calomel electrode.

## Data Availability

Not applicable.
